# The Treatment of Complementary and Alternative Medicine on Premature Ovarian Failure

**DOI:** 10.1155/2021/6677767

**Published:** 2021-04-15

**Authors:** Jing Lin, Denghui Wu, Liyan Jia, Mengmeng Liang, Siyu Liu, Zhen Qin, Jiao Zhang, Yanhua Han, Songjiang Liu, Yuehui Zhang

**Affiliations:** ^1^Chinese Medicine Department, The Second Affiliated Hospital of Harbin Medical University, Harbin, China; ^2^Heilongjiang University of Chinese Medicine, Harbin, China; ^3^First Affiliated Hospital, Heilongjiang University of Chinese Medicine, Harbin, China; ^4^Second Affiliated Hospital, Heilongjiang University of Chinese Medicine, Harbin, China

## Abstract

It has been confirmed by growing evidence that common hormone replacement therapy is associated with an increasing risk of causing cardiovascular disease and cancer, while complementary and alternative medicine (CAM) is gaining popularity and application in more and more patients with premature ovarian failure (POF). Although there is little data concerning the clinical safety and efficacy of CAM, the literature includes application studies on the phytoestrogen-rich herbal, acupuncture treatment and intervention therapy. This article reviews recent literature on CAM therapy for POF, aiming to provide theoretical support for clinical application.

## 1. Instruction

Premature ovarian failure (POF) refers to a condition in women who are in the normal range of menarche age and the normal development of secondary sexual signs before the age of 40 with ovarian dysfunction or even failure. The hormone is characterized by high gonadotropin and low estrogen, especially FSH, FSH > 40 u/L [1]. Moreover, the main clinical manifestations are menstrual cycle disorders, amenorrhea, fertility decline, and even infertility. At the same time, it is also paralleled with hot flashes, night sweats, insomnia, psychological distress, and sexual dysfunction that are similar to the symptoms of menopausal transition. The reduction of estrogen levels can also increase the incidence of osteoporosis, ischemic heart disease, autoimmune diseases, Alzheimer's disease, etc., as well as the risk of death, bringing many serious consequences [2–4]. The incidence of POF in patients over 40 years old is about 1% and over 30 years old is about 0.1% [1]. With the enlargement of social pressure, the incidence of POF is on the rise, and the age of onset is also younger than before. The exact etiologies of POF are still unclear, and most of them are believed to be related to iatrogenic factors, chromosomal/genetic defects, autoimmune diseases, infections, environmental factors, congenital enzyme deficiency, idiopathic factors, etc. [2, 3, 5–7].

Most of the symptoms of POF are associated with estrogen deficiency; therefore, the general treatment in western medicine of POF is hormone replacement therapy (HRT) simulating physiological hormone release. HRT can relieve perimenopausal symptoms and reduce the incidence of ischemic cardiovascular disease, osteoporosis, urinary symptoms, and Alzheimer's disease, improving the quality of life and prolonging life [8, 9]. POF patients cannot normally ovulate mature follicles in order to naturally conceive because of the ovarian follicle depletion; however, egg donation is a common method for treating POF infertility and the in vitro activation (IVA) and bone marrow-derived stem cells also have potential in treating POF infertility [10–12]. Additionally, melatonin supplement therapy is also playing an active role in preventing and treating ovarian dysfunction caused by chemotherapy [13]. Although HRT can relieve some clinical symptoms, it is not effective in restoring ovarian function and fertility and may increase the risk of ovarian cancer, breast cancer, endometrial cancer, thrombotic disease, meningioma, and other diseases [14–18]. Therefore, it is necessary to find an alternative therapy to supplement or replace the conventional western medicine treatment in order to reduce the adverse reactions brought by the conventional western medicine treatment.

CAM is defined as “a group of diverse medical and healthcare systems, practices, and products that are not generally considered part of conventional medicine” [19]. The National Center for Complementary and Alternative Medicine (NCCAM) classified CAM treatments into five major categories: traditional medical practices, such as whole medical systems; mind-body interventions; biological substance–based practices; manipulative and body-based practices; and energy medicine. The use of CAM is related to many factors such as patients' ethnic education, perceived behavior, positive attitude, credibility, and cultural embedment [20–22]. The global usage rate of CAM reaches 9.8%–76.0%, varying in different countries. In the United States, the adult usage rate is 38%, while in Trinidad and Tobago the usage by nurses is as high as 92.4% [20].

Complementary medicine refers to methods used with conventional medicine; on the contrary, alternative medicine refers to methods that could replace conventional medicine. CAM may be beneficial to women who have failed or have contraindications to conventional treatment [23]. It has played a great role in the treatment of gynecological diseases, and some CAM therapies have great value in the treatment of breast cancer [24]. The use of CAM in the treatment of endometriosis can effectively shrink the lesion, inhibit the symptoms, and reduce the recurrence rate [25]. CAM can improve menstruation and ovulation in patients with POF and at the same time improve perimenopausal symptoms such as hot flashes, sweating, insomnia, anxiety, and osteoporosis. Consequently, the treatment of POF with CAM is a problem worthy of discussion. This paper reviews the efficacy of CAM in the treatment of POF by means of retrieval of literature on herbal therapy, acupuncture, massage, psychotherapy, dietary supplementation, vitamin, calcium, exercise therapy, bioelectric therapy, and other treatments.

## 2. Herbal Remedies

The term “herbal” is derived from the Latin “herba,” translated as “grass.” It is one of the oldest healthcare methods and made a great difference in ancient times. Plants have long been recognized for their therapeutic properties. Indigenous cultures around the world have used traditional herbal medicine to treat a myriad of maladies [26]. In the western world and many Asian countries, including China and India, patients often use complementary medicines in the form of herbal remedies. Herbs are widely used in the treatment of POF in East Asian countries, and herbal medicine is also widely practiced in clinics in China [27]. Herbal medicine is one of the most common forms of traditional Chinese medicine (TCM) and has always been considered the best among the treatment methods in Chinese medicine. Studies have shown that CHM is effective in treating POF [28]. There are numerous reports in the Chinese literature about the treatment of POF with herbal remedies, but only abstracts are available in English. TCM's holistic theory, “pattern identification,” diagnoses symptoms based on diseases and development and treats them based on individual needs. The concept of “kidney” takes a basic role in the physiology of female menstruation and pregnancy in TCM's theories; most clinical physicians also believe that kidney deficiency is the main etiology and pathogenesis of the disease. The basic method of treating POF is to invigorate the kidney; commonly used methods include invigorating the kidney, promoting blood circulation, and invigorating the kidney and spleen, which are confirmed effective through modern research on the treatment of POF [29–31].

### 2.1. Compound Chinese Medicine

Through experiments and clinical experiences, Chinese clinical workers have found that TCM prescription of the POF treatment has many advantages, with effect being significant [32–34]. Furthermore, a section of researchers found that Bushen Huoxue decoction can effectively improve the clinical symptoms of patients with POF, by adjusting FSH levels and FSH/LH ratios, increasing AMH levels, and adjusting ANA-ACA-AOA, ACT-INH-FS, and other pathways to alleviate symptoms through clinical randomized controlled trials (RCT). Zuogui pill (ZGP) is a classic prescription of TCM, which has been widely used in the treatment of POF. Although there is no prominent difference in pregnancy rate after receiving ZGP treatment, the oestrus cycle, ovarian ultrastructure, follicular number, and corpus luteum are significantly improved. ZGP resulted in a significant decrease in serum follicle-stimulating hormone (FSH) concentration and an increase in estradiol (E2). In addition, after administration of ZGP, a significant downregulation of Bax and cytochrome c (Cyt-c) and an upregulation of B-cell lymphoma/leukemia 2 (Bcl-2) were observed at both the gene and protein levels; thus, it is assumed that the effect may be achieved by inhibiting mitochondrial-dependent apoptosis in follicles [35]. Moreover, Bushen Shugan decoction, Yulinzhu, Guilu Erxian decoction, Bushen Tiaojing decoction, and Bazhen decoction have been also proven to have therapeutic effects on POF (Tables 1–3) [36–44].

### 2.2. The Monomer of Herbal Medicine

#### 2.2.1. *Cistanche*

In addition to the prescriptions, single herbal medicine is also effective in the treatment of POF. *Cistanche salsa* is the dry fleshy stem with scaly leaves of *Cistanche* of Orobanchaceae family. It is an extremely valuable TCM which is known as “desert ginseng.” The use of *Cistanche salsa* in medicine was first seen in Classic of *Shennong Materia Medica*. During the Northern and Southern dynasties, *Cistanche salsa* was used in dietotherapy to tonify deficiency with the functions of tonifying kidney yang, tonifying essence and blood, moistening intestines, and purging bowels; thus, it was often used in the treatment of impotence and infertility [45]. The main chemical components of *Cistanche salsa* are phenylethanoid glycosides, iridoid, lignan, polysaccharide, etc. and are also rich in phytoestrogens. Modern pharmacological studies have shown that it has many biological activities, for instance, antiaging, protecting liver, relieving physical fatigue, antiosteoporosis, and moistening bowels [46]. Pan et al. [47] discovered through animal experiment that the decoction of *Cistanche* could improve the POF caused by cisplatin by interfering with the apoptosis of ovarian granulosa cells induced by cisplatin, which is of therapeutic significance to POF after chemotherapy. *Cistanche* can reduce follicular atresia and apoptosis, by regulating the level of sex hormones and inhibiting the expression of TNF-*α* and IFN-*γ*, and then slow down the rate of ovarian failure. *Cistanche* deserticola can also upregulate Bcl-2/Bax. It is suggested that *Cistanche* deserticola can inhibit POF, and its mechanism may be related to the level of sex hormone and the expression of TNF- *α*, IFN- *γ*, and apoptosis-related protein Bcl-2/Bax in ovary [48].

#### 2.2.2. *Cuscuta*

*Cuscuta australis* R.Br or *Cuscuta chinensis* Lam. is the dry mature seed of the southern *Cuscuta australis*. This product mainly contains triterpenoid acids, sugars, saponins, starch, etc. [49]. The functions of *Cuscuta* basically include tonifying liver and kidney, consolidating concentration and shrinking urine, strengthening muscles and bones, promoting saliva and thirst quenching, and tranquilizing spirit [50]. The total flavonoids from dodder (TFSC) can significantly restore the ovarian function of rats with POF, increase the ovarian weight and the number of follicles, raise estrogen levels, and have an obvious effect on POF [51]. The experiment of Li et al. [52] proved that a variety of active ingredients extracted from dodder-*Lycium barbarum* drugs may have an effect on treating POF through the PI3K/AKT signaling pathway, MAPK, and other pathways, and dug out the potential active ingredients like sterol, sesamin, and potential target IL-6 and TNF simultaneously.

#### 2.2.3. *Epimedium*

*Epimedium*, as one of the medicinal plants, comes from Berberidaceae, perennial herbs, also known as Xianling spleen. Compendium of Materia Medica believed that it has the effect of invigorating qi, strengthening muscles and bones, tonifying waist and knee, strengthening heart, etc. *Epimedium* is often used to treat female infertility, irregular menstruation, POF, and other diseases. Herba Epimedii mainly contains flavonoids, polysaccharides, lignin, phenolic glycosides, alkaloids, and other kidney-enhancing and aphrodisiac biological active components [53, 54]. The studies of Zhang et al. [55] found that *Epimedium* is closely related to the vitamin D axis, assuming that *Epimedium* may be used to treat female reproductive system diseases by regulating the expression level of vitamin D axis. Vitamin D axis may be a potential target for Chinese Herba *Epimedium* to interfere with female reproductive system diseases.

#### 2.2.4. Maca

Maca is the rhizome of the cruciferous plant Maca solo, also known as beet root or Peruvian ginseng. Native to the Andean plateau of South America, its fleshy root is short conical and the outer skin is purple, cream, or yellow [56]. Maca mainly contains macaramide, macaenes, alkaloids, glucosinolates, thiohydantoin, sterols, and other chemical components [57], which play a role in sexual function, spermatogenesis, female reproductive function, memory, depression, anxiety, energy, benign prostatic hyperplasia, osteoporosis, and metabolic syndrome [58]. The experiment conducted by Wang et al. [59] used POF model of kidney yang deficiency syndrome in SD rats with domestic Maca, imported Maca from Peru, and evaluated the effect and mechanism of Maca in warming kidney yang and maintaining ovary. It was concluded that both kinds of Maca could improve kidney yang deficiency syndrome and maintain ovary in SD female rats, being effective after two weeks of Maca and more obvious after four weeks. The mechanism by which Maca can improve the maintenance of ovary of kidney yang deficiency syndrome is related to the effect of warming and tonifying kidney yang, invigorating spleen, and tonifying qi and has biaxial regulation on kidney-Tiangui-Chongren-cell axis and hypothalamus-pituitary-ovary-uterus reproductive axis.

#### 2.2.5. Muniziqi

Muniziqi, a Chinese medicine, has the effect of preventing and treating POF and effectively changing the tissue morphology and hormone level of gonadal axis. Some studies believed that [60] chronic stress can lead to POF. Histopathological changes of the ovary and hormonal disorders (E2, FSH, LH) may be among the important mechanisms. Muniz can improve the early lesions of POF, and its pharmacological mechanism may be related to the regulation of the disordered hormone levels (E2, FSH, LH) and the downregulation of the expression of related proteins PFN1 and CFL1 [61].

## 3. Acupuncture and Moxibustion

In recent years, acupuncture and moxibustion therapy has been widely used in the clinical treatment of various gynecological diseases due to its good clinical efficacy and few side effects [62, 63]. Since the pathogenesis of POF is mostly related to liver, spleen, kidney, and blood essences, acupuncture and moxibustion therapy can dredge meridians, regulate qi and blood, balance yin and yang, and harmonize the functions of viscera. Research has shown that acupuncture can regulate the hypothalamic-pituitary-ovarian axis (HPOA) function by activating the dopamine system in the brain, adjusting the function of HPOA so as to restore the dynamic balance of reproductive endocrine system back to normal physiology. Moreover, it is also the most promising treatment method to improve menopausal symptoms and reduce serum follicle-stimulating hormone and luteinizing hormone levels [63, 64]. Acupuncture and moxibustion therapy have been fully recognized in POF treatment and have proved to be superior to western medicine [64, 65]. According to the clinical study of acupuncture and moxibustion for primary ovarian insufficiency (POI), the intervention methods of acupuncture and moxibustion include filiform needle, electroacupuncture, acupoint catgut-embedding therapy, padding moxibustion, moxibustion, and auricular point sticking therapy [66].

### 3.1. Acupuncture

#### 3.1.1. Acupoints

According to the statistics, the proportion of each type of POF from high to low in proper order is as follows: disharmony of lesser yin, disharmony of lesser yin and reverting yin, disharmony of lesser yin and greater yin, disharmony of greater yin, and disharmony of reverting yin [67]. This indicates that deficiency of kidney essence is the key to POF; qi dynamic stagnation and dual deficiency of qi and blood are also the main factors. Therefore, these commonly used acupoints should have the functions of tonifying the kidney, nourishing the liver and invigorating the spleen, and nourishing the three yin meridians and blood. Acupuncture and moxibustion acupoints of POF are mostly distributed in the back, lower limbs and abdomen [68]. Shenshu (BL23), Pishu (BL20), and Ganshu (BL18) of Foot Taiyang Channel of Bladder are commonly used in clinic on the acupoints of nape. Acupoints of the lower limbs such as Sanyinjiao (SP 6), Xuehai (SP 10), and Yinlingquan (SP 9) in Spleen meridian; Taixi (KI3), Yongquan (KI1), and Fuliu (KI7) in kidney meridian; and Zusanli (ST36), Fenglong (ST40), and Shangjuxu (ST37) in stomach meridian are mainly used [69]. Guanyuan (RN4), Zhongji (RN3), Qihai (RN6), etc. in Conception Vessel are the main used points in clinic [67, 70, 71]. Among them, acupoints of the lower limbs were used most frequently, accounting for 33.92%, and lower back was used most frequently, accounting for 32.76%. Among all the acupoints, Sanyinjiao (SP6), Guanyuan (RN4), and Shenshu (BL23) ranked the top 3, with the support degrees of 62.65%, 57.83%, and 49.40% [72].

#### 3.1.2. Acupuncture Method

Acupuncture has its unique advantages in treating POF because of its various treatment methods. Zhang et al. [73] showed that acupuncture has certain advantages in the treatment of POF and can achieve a similar effect to estrogen; its mechanism may be related to the upregulation of gene and protein expression in the PI3K/Akt/mTOR signaling pathway. Yao et al. [74] randomly divided 104 patients with POF into acupuncture group and western medicine group. The results showed that the total effective rate of the acupuncture group was 90.4%, which was higher than the western medicine group's 67.3%; the acupuncture group's FN-*γ* and expression of TNF-*α* were significantly lower than those of the western drug group (*P* < 0.05). It effectively adjusted the levels of serum LH, FSH, and E2 and improved the pituitary and ovarian endocrine in patients with POF. Wu et al. [75] reported that the menstrual recovery rate of POI patients treated with Fang's regulating menstruation and pregnancy promoting acupuncture method is 86.7%, which can effectively adjust the menstrual cycle of patients with early-onset ovarian insufficiency, increase endometrial thickness, effectively reduce serum FSH levels, and improve symptoms of ovarian function and low estrogen. Zhang et al. [76] and Guo et al. [77] found that the acupuncture is superior to hormone therapy in improving clinical symptoms and menopausal recurrence and reducing serum FSH and LH levels in patients with POF. In addition, the latter can significantly increase the rate of menstruation, improve the clinical effective rate, and create better conditions for pregnancy, and the curative effect is stable. Wang et al. [78] adopted a prospective case sequence study design and used preacupuncture intervention to interfere with POF [79]. The results showed that the levels of FSH, LH, and E2 in patients were adjusted and improved in varying degrees, and the total effective rate after treatment was 86.7%. The clinical pregnancy rate of the latter reached 31.8%. In the study of Shang et al. [80], it was concluded that acupuncture can improve the serum hormone level of patients with early-onset ovarian insufficiency. There is a correlation between menstruation and pregnancy outcome in patients with POI. The possibility of pregnancy in patients with oligomenorrhea is higher than that in patients with amenorrhea, and acupuncture can improve the level of estrogen in obese patients more obviously.

#### 3.1.3. Acupuncture Point Embedding Therapy

As the development and extension of acupuncture therapy, acupoint catgut-embedding therapy is an improved needling method relying on the needle retaining theory [81]. On the basis of inheriting the short-term acupuncture effect, while strengthening the sense of acupuncture and prolonging the action time, it has been paid more and more attention in the treatment of gynecological diseases. Li et al. [82] through clinical randomized controlled study found that the acupoint catgut implantation combined with artificial periodic therapy achieved remarkable improvements in the clinical symptoms of POF in patients. Acupoint embedding could effectively improve estradiol level [83]. In the experiment conducted by Chen et al. [84], 18 patients with POF were given acupoint catgut-embedding therapy, with an average treatment period of 6 months and total effective rate of 72.2%. Studies have shown that acupoint catgut-embedding therapy combined with Kuntai capsule [85] and that combined with artificial menstrual cycle therapy [86] can be filled with kidney essence, promote the transformation of Tiangui, promote the prosperity of Chong and Conception Channels, and further coordinate the “kidney-Tiangui-Chongren-Baogong” axis. It can regulate the hormone level in ovary and promote the recovery of ovarian function. In Chen et al.'s clinical study [87] on the treatment of POF by combining TCM for tonifying the kidney and soothing the liver with acupoint catgut embedding, it was suggested that this combination had accurate efficacy, low recurrence rate, and few adverse reactions. In addition, in the experimental study conducted by Xia et al. [88] on the decline of ovarian reserve function and POF in female rats treated by acupoint cat embedding and TCM, the results suggested that cat embedding and TCM have the effect of improving the ovarian reserve function and have a positive effect on the prevention of the decline of ovarian reserve function and the treatment of POF.

#### 3.1.4. Ventro-Acupuncture

Ventro-acupuncture is a microneedle therapy to treat systemic diseases by acupuncture at abdominal acupoints. Regarding the regulation of the Shenque system as the core, it treats diseases by regulating the body's meridians and nervous system and regulating the balance of visceral functions. Li et al. [89] used “Zhang's abdomen three needles” to treat POF. The total effective rate of the treatment group was 93.33%, which achieved definite curative effect. Cao et al. [90] observed the clinical efficacy of Guishen pills combined with ventro-acupuncture in the treatment of POF. Compared with the control group, the treatment group had better efficacy. At the same time, the treatment group was excellent in the improvement of E2, FSH, and LH.

#### 3.1.5. Auricular Acupuncture

Auricular acupuncture is a traditional Chinese meridian therapy, including ear embedding therapy, ear sticking therapy, auricular bleeding, and automatic ear acupuncture therapy equipment. It achieves the purpose of preventing or curing diseases by stimulating auricular acupoints. Luo et al. [91] conducted a systematic review and meta-analysis of the efficacy and safety of ear acupuncture in the treatment of POI by searching multiple databases before August 2020. The results showed that stimulation of specific ear points can enhance the physiological functions of specific parts of the human body, promote qi and blood circulation, and regulate the reproductive function of the hypothalamus-pituitary-ovarian axis. Yang et al. [92] explored the clinical effect of body acupuncture combined with ear acupuncture in the treatment of kidney deficiency and liver depression POF; the total effective rate in the treatment group was 80%, which was superior to 66.67% in the control group. Meanwhile, the ear bean combined TCM therapy is widely applied in clinical practice. For example, Yang et al. [93] selected 90 patients with POF and divided them into 3 groups: 30 cases in Chinese medicine group, 30 cases in auricular group, and 30 cases in Chinese medicine combined with auricular group, with three menstrual cycles as a course of treatment and three courses of consecutive treatment. The results showed that the total effective rate for the auricular point combined with CHM decoction was 90.00%, for the Chinese medicine treatment group was 83.33%, and for the auricular acupoint treatment group was 76.66%. In the experiment conducted by Shen et al. [94], selected 60 patients were divided into two groups including Guishen pill with auricular point treatment group (combined treatment group) and artificial cycle group, with 30 patients in each group. Compared with the artificial cycle group (80.0%), the total effective rate (93.3%) of the treatment group was significantly higher (*P* < 0.05). Jin et al. [95] selected 70 patients with POF of kidney deficiency and liver depression type and then randomly divided them evenly into treatment group and control group, with 35 cases each; the control group was administered Marvelon orally, while the treatment group was treated with Bushen Tiaojing decoction combined with auricular acupoint pressing. The result showed that the total effective rate of the treatment group was 84.85% after 3 months of treatment, while that of the control group was 52.94% with significant differences.

#### 3.1.6. Electroacupuncture

Electroacupuncture (EA) therapy uses density wave, and its low-frequency pulse current stimulates acupoints through filiform needle. It can adjust human body function, promote blood circulation, and stimulate muscles and nerves. Zhang et al. [96] found that the effect of EA on PI3K/Akt/mTOR signaling pathway may be one of the mechanisms involved in the attenuation of POF in mice. Tan et al. [97] first estimated that transcutaneous electrical acupoint stimulation (TEAS) can be used as a potential therapy to reduce radiation-induced ovarian failure by inhibiting the loss of primordial follicles, increasing the secretion of serum AMH, and inducing antioxidant and antiapoptotic systems. In clinic, electroacupuncture and heat sensitive moxibustion are also used to treat premature ovarian failure. Heat sensitive moxibustion is a kind of therapy guided by meridian theory. It uses burning moxa stick to moxibustion heat sensitive acupoints to stimulate acupoints to produce heat sensitive moxibustion feeling. Moxibustion pays attention to qi sensation, dredges meridians, regulates viscera, and improves curative effect. Tian et al. [98] treated 60 patients with POF of kidney deficiency and liver depression type by electroacupuncture combined with heat sensitive moxibustion, with a total effective rate of 93.3%.

### 3.2. Moxibustion

Moxibustion refers to a kind of external treatment method that uses moxa leaf and other inflammable materials or drugs to burn or fumigate and iron acupoints or affected area after being ignited, so as to achieve the purpose of disease prevention and treatment through its warm stimulation and drug effect. Studies have shown that there is no significant difference in the clinical efficacy of different moxibustion methods in the treatment of POF, but all of them can be used for treating POF because of their safety and efficacy [99].

#### 3.2.1. Warm Acupuncture

Warm acupuncture is a method of combining acupuncture and moxibustion. It can simultaneously adjust viscera and bowels, warm qi and blood, and balance yin and yang with the help of filiform needle, drug, and heat through the conduction of meridians and collateral. Wu et al. [69] study found that warm acupuncture at Zusanli (ST36) and Guanyuan (CV4) combined with ginger moxibustion at Baliao point had significant effect on POF patients and on the improvement of FSH/LH, PSV, and AFC. Clinically, warming acupuncture combined with traditional Chinese medicine decoction has achieved good curative effect on POF. Wei et al. [100] studied the effect of Yikun Tiaojing decoction combined with warm acupuncture on POF and its influence on ovarian blood flow state. Patients in each group were randomly divided into observation group and control group. All patients were given hormone therapy. The control group was given warm needling on this basis, and the observation group was given Yikun Tiaojing decoction on the basis of the control group. With continuous medication for 28 days as a course of treatment, two groups of patients were treated for 3 courses. The treatment effect, ovarian artery blood flow status, sex hormone levels, and ultrasonic examination indexes were compared between the two groups. The results showed that the total effective rate of the control group was 73.53% and that of the observation group was 94.12%. It was concluded that the observation group was significantly better than the control group.

#### 3.2.2. Umbilicus Moxibustion

Umbilicus moxibustion combines the advantages of moxibustion, drugs, and acupoints, which can stimulate, mobilize, and strengthen the body's own regulatory system; promote the blood circulation of uterus and ovary; and promote the development and maturation of follicles. Yu et al. [101] discussed the clinical efficacy of umbilical acupuncture combined with Hunyuan moxibustion in the treatment of POF. 90 patients with POF were randomly divided into research group and control group; the research group was treated with acupuncture at navel, and Hunyuan moxibustion was performed at Shenque point after the acupuncture. The control group was given hormone cycle replacement therapy. The total effective rate of the research group was 93.33% compared with 71.11% of the control group. It has achieved good results in rat experiments and has considerable development prospects. In the study conducted by Jia et al. [102], they observed the effect of drug-separated moxibustion on the expression of Bcl-2 and Bax in rats with POF and the effect on the expression of VEGF and AMH in rats with POF [103], and they found that drug-separated umbilical therapy had significant promoting effects on the number of follicles and ovarian reserve function in the rats with POF and could also improve the ovarian function.

## 4. Other Treatments for POF

### 4.1. Massage

In clinical practice, traditional Chinese medicine combined with massage has quite good curative effect. For example, the experiment conducted by Liu et al. [104] discussed the clinical efficacy of Bushen Tiaojing decoction combined with acupoint massage therapy, the 60 patients were randomly divided into A and B groups, and patients in both groups were treated with artificial cycle hormone replacement therapy. On that basis, group B was treated with Bushen Tiaojing decoction and acupoint massage therapy, and the therapeutic effect and serum sex hormone levels of the two groups were compared. The results showed that the total effective rate of B group was higher than that of A group, the levels of FSH (follicle-stimulating hormone) and LH (luteinizing hormone) in B group were lower than those in A group, and the E2 (estrogen) levels in B group were higher those that in A group. Wu et al. [105] observed the clinical efficacy of Yishen Huoxue decoction combined with massage in the treatment of 33 patients with POF in the treatment group taking self-made Yishen Huoxue decoction orally, conducting acupoints (Dazhui (DU14), Tao Dao (DU13), Shenzhu (DU12), Lingtai (DU10), Yongquan (KI1), Zigong (EX-CA1), Sanyinjiao (SP6), and Zusanli (ST36)) massage therapy. In the control group, 26 patients were given Tibolone tablets once a day, and 8 mg of progesterone was added after 21 days for five days; both groups were treated for 6 months. Changes of E2, FSH, and LH level and improvement of clinical symptoms were observed before and after treatment. The results suggested that there was significant difference between the two groups before and after treatment, and the curative effect of the treatment group was more obvious than that of the control group.

### 4.2. Psychotherapy

POF has a negative effect on the quality of life and psychological well-being of patients. The modern integrative approach to the treatment of disease includes biological, modern ways of treating diseases with the integration of physical, mental, biological, psychological, and social factors, which may make people more inclined to promote or continue the medical efficacy. It is not assumed that there is a direct causal relationship between mental and physical phenomena, but that biological, psychological, and social factors influence each other at the same time [106, 107]. Recently, through clinical trials [108], some scholars have observed that the cure rate of patients with POF is significantly increased due to the combination of conventional medical treatment and psychological treatment, which can not only improve the clinical symptoms, but also reduce the subjective burden and mental pressure and improve the quality of life. A large number of clinical experimental studies have shown that [109, 110] psychological nursing of patients with POF can significantly improve the adverse mood of the patients, play a good role in promoting the treatment effect, and have clinical promotion and application value. More and more doctors realize the benefits of adapting the combination of biopsychosocial therapy to treat diseases, and combine psychosomatic therapy with modern medical treatment based on their clinical expertise, which may greatly contribute to the treatment and prognosis of POF.

### 4.3. Dietary Supplements

TCM has always believed that medicine and food are homologous, having the same use and effect. A considerable part of common clinical medicine is not only medicine but also food. In the long development of TCM, food therapy has always attached great importance to nutrition. Zhang Xichun, a famous modern doctor, once said, “Diet therapy can not only cure diseases but also satisfy one's hunger. What's more, the diet therapy is more palatable than the traditional drugs, and it is believed that diet therapy is of no harm for treating disease, which means that if the chosen diet therapy is matched with the disease, the time of recovering from disease will be shorten, even if it is not, the disease will not get worse.” Chinese medicine scholar Doctor Wei [111] followed the ancient admonition, repeatedly practiced, carefully selected the medicine bait, and made a dietetic prescription by himself. The prescription is based on four kinds of medicine, namely, Nansha ginseng, yam, lotus seeds, and *Dendrobium candidum*. The blood deficiency person removes *Dendrobium* and *Angelica sinensis*; qi deficiency person adds *Astragalus membranaceus*. All the carefully selected medicines are food and medicine. Stewing the medicine together with the tube bone, the soybean, the radish, and the mushroom has the guiding significance and the practical value in the clinical treatment of POF. In a model study conducted by BaezaI et al. [112], a polyphenol-supplemented diet (from flavonoids such as soybeans and green tea) was used to treat sham surgery and ovariectomized mature mice. It was found that dietary polyphenols can be used to restore immune function in elderly mice [113]. Some nutrients are protective factors of ovarian function like legume food, which can reduce the level of blood follicle-stimulating hormone in women and thus protect ovarian function. So far, there is little research on the relationship between dietary nutrition and POF which means that further research is needed to determine the risk factors that may affect the incidence of POF in dietary nutrition, thus guiding the rational diet structure, so as to prevent the occurrence of POF to a certain extent. Although there are limited studies on the efficacy of dietary supplements in POF, the results of these studies show promising evidence.

### 4.4. Vitamin Supplement Therapy

Vitamin D is a steroid hormone which is mainly produced by the skin after being exposed to sunlight while slightly taken in food, combining with calcium to relieve osteoporosis caused by POF. Vitamin *D* is found naturally in some food, such as certain fish (salmon, herring, and sardines), egg yolks, and mushrooms [114]. Many scholars have studied the relationship between serum vitamin *D* level and the influence of ovarian-related diseases [115–118], showing that vitamin *D* concentration in POF patients is lower than that in healthy people [119]. The experiment of Chang et al. [120] showed that vitamin *D* may enhance ovarian function by regulating androgen activity to treat POF. Most experts agree that vitamin *D* supplementation is necessary when the serum concentration of vitamin *D* is below 50 nmol/L (the normal range is 125 nmol/L), especially in women with diminished ovarian reserve undergoing [121]. Cheng et al. [122] studied the combination of Zishen Buyi Shujing decoction with vitamin E in the treatment of POF, which can effectively relieve clinical symptoms, promote menstrual cycle recovery, and help to improve the level of serology.

### 4.5. Calcium

POF leads to a decrease in estradiol concentration and an accelerated loss of bone density [123]. In the case of bone density loss, nondrug therapy and lifestyle changes are first-line interventions. The National Osteoporosis Foundation strongly recommends several ways to keep bones healthy. Quitting smoking, weight-bearing, strengthening muscle exercise, avoiding excessive alcohol consumption, and properly supplementing calcium and vitamin *D* [124] are the main living principles. WHO guidelines indicate that 1,000 mg of calcium per day is sufficient to maintain the appropriate bone density, while consumption of more than 1,200 to 1,500 mg per day may increase the risk of renal calculus or cardiovascular diseases, with no positive effect on bone mass. With regard to vitamin *D*, an intake of 800 IU per day is sufficient and vitamin *D* supplementation should be controlled under 25(OH)D (the recommended serum concentration is above 30 ng/ml) [124, 125].

### 4.6. Exercise Therapy

Exercise combined with progressive relaxation training can effectively improve the mental health status of patients with POF as it is simple and easy to implement without increasing the economic burden of patients, with high patient acceptance and strong operability [126]. The application value of exercise therapy and psychological counseling in patients with POF is extremely high, with obvious improvement of symptoms, normal sex hormone level, improvement of psychological state, and high satisfaction, which is worthy of promotion and extension [127]. Ana et al. [128] studied the effects of angiotensin-converting enzyme (ACE) inhibition and aerobic exercise on the heart of elderly female rats with POF (10 weeks) and found that physical exercise and electromagnetic therapy had similar beneficial effects on the morphology and heart function of elderly female rats with POF. Although the reason still remains a question, both treatments promote a reduction in myocardial contractile power, and the reduced hydroxymethyl-epinephrine sensitivity suggests that both treatments may reduce the sympathetic action of the heart. In conclusion, the clinical effect will be more significant when the exercise therapy is combined with other therapies, especially the psychological intervention therapy.

### 4.7. Biomimetic Electrotherapy

With the continuous improvement of medical level, pelvic floor rehabilitation technology has been introduced in China and gradually used in clinical practice, becoming an important treatment in gynecology at the present stage step by step. The application of pelvic floor EMG imitating bioelectric stimulation in the decline of ovarian reserve function is gradually known. The principle [129] is to strengthen the whole pelvic floor muscle group by stimulating the electrode placed in the perineum at different frequency, stimulate the innervation of the pelvic floor muscle, and restore the nerve function. Li et al. [130] explored the clinical effect of TCM combined with biomimetic electrical stimulation on patients with decreased ovarian reserve function. The results showed that it could effectively regulate the endocrine and ovarian related indexes of the patients, improve the decline and even deficiency of estrogen in patients, at the same time significantly improve the symptoms of bone metabolism and osteoporosis, and reduce the physical and mental pain, anxiety, and depression of patients in order to achieve the purpose of enhancing the quality of life. Guo et al. [131] retrospectively analyzed the clinical data of 80 patients with POF in order to explore the effect of biomimetic electrical stimulation combined with sequential therapy of estrogen and progesterone in the treatment of POF. Studies have shown that biomimetic electrical stimulation is effective in patients with POF on the basis of sequential therapy of estrogen and progesterone, which can further promote the improvement of sex hormone level and bilateral ovarian hemodynamics, and does not increase the risk of adverse reactions with good safety.

Bionic electrical stimulation therapy is a kind of physical therapy, which is safe to use and easy to operate and has high compliance. It can be used as an adjuvant treatment for patients with premature ovarian failure, and its exact mechanism needs to be further explored.

Apart from that, many other treatments such as hypnosis, meditation, and practice have also proved to have good curative effects in gynecological diseases including dysmenorrhea [132, 133], ovarian cancer [134], endometriosis [25], and pelvic inflammation [135], but the curative effect in POF remains to be further studied and observed.

## 5. Discussion

In conclusion, although the effectiveness of herbal therapy and acupuncture therapy has been well documented, it is difficult to make definitive recommendations for clinical guidance. Most of these studies are hindered by a small sample size or limited to one ethnic group, and there are few studies on adverse reactions, so it is not clear whether the effects of these treatments span all ethnic groups and nationalities. Therefore, before making any clinical recommendations, it is necessary to conduct a large number of clinical randomized controlled trials, entirely composed of POF participants with different ethnic backgrounds. Existing studies have shown that interventions such as psychotherapy, exercise therapy, and dietary supplement therapy can reduce the related symptoms caused by POF, and there are almost no adverse reactions, which seems to provide safe treatment options worth considering; however, due to the limited number of studies, the specific treatment mechanism is not clear and there is lack of empirical support.

CAM is an increasingly mature treatment system. In future studies, more high-quality randomized controlled trials are needed for each CAM intervention to make the results more reliable and professional. There have been a large number of studies on herbal medicine and acupuncture therapy, and the treatment is relatively mature. Randomized controlled trials based on standardized preparations should be used in a consistent way to conduct a more systematic and professional review of these interventions and meta-analysis of the results, so as to show that they have clinical efficacy value. POF can be accompanied by mild or severe sexual dysfunction, eventually leading to depression or infertility, so it is more important for patients with POF to correctly dredge their psychology on the basis of respecting their privacy. Therefore, we suggest that, in today's fast-paced life, we should fully respect women, especially the lover, whether in life or work to give his wife tolerance and love of encouragement.

In the treatment of POF, in addition to mainstream medicine, CAM will continue to be widely used to better control the physical and mental diseases caused by POF, including preventing deterioration, reducing symptoms, and providing a healthy lifestyle. However, further clinical studies are needed to evaluate the effectiveness, safety, and cost-effectiveness of CAM in POF. A large number of complex genetic mutations and the interaction of today's fast-paced living environment play an important role in POF. CAM therapy combined with conventional therapy is expected to find a new treatment strategy for POF.

## Figures and Tables

**Table 1:** The summary of compound Chinese medicine on POF.

Classical prescription of TCM	Components	References
Bushen Huoxue decoction	*Codonopsis* 20 g, danshen root 20 g, *Angelica* 20 g, *Astragalus* 20 g, *Epimedium* 15 g, *Morinda* 12 g, *Rehmannia* 12 g, *Cuscuta*, *Rubus idaeus* 12 g	[32]
Zuogui pill (ZGP)	*Rehmannia* 20 g, yam 15 g, wolfberry 15 g, *Cornus alba* 10 g, *Cuscuta* 10 g, Lujiao 10 g, testudo gum 10 g, *Codonopsis* 20 g, *Ligustrum vulgare* 20 g, *Eclipta prostrata* 20 g, Chuan *Achyranthes* 10 g, *Angelica* 10 g, Xiangfu 10 g, *Caulis spatholobi* 20 g, turmeric 10 g	[36]
Guilu Erxian decoction	Testudo gum 6 g, Gum cornibus extant 6 g, wolfberry 10 g, *Angelica* 10 g, yam 15 g, Taizishen 15 g, *Cuscuta* 15 g, arbor aglaophotis 10 g, album aglaophotis 10 g, *Cornus* 10 g, *Rubus idaeus* 15 g, teasel 10 g, *Rehmannia* 10 g, *Ligustrum vulgare* 10 g, semina pirorum 10 g, Flumen purpura Car 6 g	[37]
Bushen Shugan decoction	*Bupleurum falcatum* 12 g, *Angelica* 15 g, Frixum album aglaophotis 12 g, *Cuscuta* 15 g, *Atractylodes fricta* 12 g, *Rehmannia* 15 g, motherwort 15 g, danshen root 12 g, licorice 6 g, gingiberi 6 g	[38]
Yulin decoction	Taizishen 15 g, *Cuscuta* 15 g, *Rehmannia* 10 g, *Atractylodes fricta* 10 g, *Poria* 10 g, *Cnidium* 3 g, *Angelica* 10 g, *Ligustrum vulgare* 15 g, *Eclipta prostrata* 15 g, *Eucommia* 10 g, *Polygonatum* 10 g, Chuanxiong 5 g, Cnecos 5 g,	[39]
Bushen Tiaojing recipe	*Rehmannia* 15 g, *Cornus* 10 g, yam 15 g, wolfberry 12 g, *Cuscuta* 12 g, *Ligustrum vulgare* 9 g, *Poria* 10 g, fleeceflower root 12 g, moro 10 g, Chuan *Achyranthes* 10 g, *Angelica* 9 g, teasel 10 g, *Eclipta prostrata* 9 g, danshen root 9 g, motherwort 10 g, arbor aglaophotis 10 g, *Cistanche* 12 g	[40]
Bazhen recipe	*Bupleurum falcatum* 6 g, *Angelica* 10 g, Chuanxiong 6 g, album aglaophotis 10 g, *Rehmannia* 10 g, *Codonopsis* 10 g, *Atractylodes* 6 g, *Poria* 10 g, licorice 3 g, Cremor cornibus extant 10 g, Xiangfu 10 g, Chuan *Achyranthes* 10 g,	[41]

**Table 2:** The summary of randomized clinical studies of compound Chinese medicine on POF.

Prescription	Design	Sample size	Interventions	Outcomes	References
Bushen Shugan recipe	RCT	*n* = 117	Control group A : Bushen Shugan recipe, control group B : artificial cycle therapy: estradiol valerate tablet + progesterone capsule, treatment group: Bushen Shugan recipe combined with artificial cycle therapy	Bushen Shugan recipe combined with artificial cycle therapy can significantly improve serum hormone levels, clinical syndrome scores, and clinical symptoms in patients with POF with kidney deficiency and liver depression syndrome.	[38]

Bushen Tiaojing decoction	Single blind, RCT	*n* = 110	Control group: hormone replacement therapy, treatment group: kidney-tonifying prescription	Bushen Tiaojing recipe can effectively reduce the symptoms and signs related to POF, improve reproductive axis status, upregulate GDF-9 and BMP-15 levels, and help to reduce the risk of long-term recurrence.	[40]

Yulin decoction	RCT	*n* = 60	Control group: sequential estrogen therapy (oral estradiol valerate tablets + progesterone capsules), treatment group: Yulinzhu	In the treatment of POI (kidney qi deficiency type), Yulinzhu could significantly improve the clinical symptoms, decrease the levels of FSH and LH, increase the level of E2, and increase the number of follicles in the basal antrum of ovary and the thickness of endometrium.	[43]

**Table 3:** The summary of basic studies of compound Chinese medicine on POF.

Prescription	Experimental type	Sample size	Interventions	Outcomes	References
Zuogui pill (ZGP)	SD rats	*n* = 54	Control group, model group, three ZGP groups (3.2, 1.6, and 0.8 g/kg), and triptorelin group	After treating with ZGP, though the rate of pregnancy showed no significant difference, the estrous cycle, ovarian ultrastructures, and numbers of follicles and corpora lutea were improved significantly.	[35]

Guilu Erxian decoction	SD rats	*n* = 40	Control group, model group, wild yam compound nutrition soft capsule group, TCM group	Compared with the control group, modified Guilu Erxian decoction can effectively increase the number of primordial follicles and primary follicles in the ovarian tissue of POI rats, and its mechanism may be related to the inhibition of ovarian miR-190 expression.	[37]

Bushen Huoxue recipe (BHR)	SD rats	*n* = 45	Normal group, POF model group, and BHR group, with 15 mice in each group	The results demonstrated treatment efficacy of BHR on POF mice and revealed that BHR might repair the dysfunction of germline stem cells in the bone marrow and thus help to improve the ovarian reserve and enhance the ovarian function of POF mice through neogenesis.	[42]

Modified Bazhen decoction, (MBD)	SD rats	*n* = 24	Control group, POF group, MBD treatment group, and Fufang Ejiao Syrup (FES) treatment group	Compared with the control group, XIAP expression was significantly lower, and miR-23a and miR-27a expression were significantly higher in the POF group. XIAP expression was significantly higher, and miR-23a and miR-27a expression were significantly lower in the MBD group. MBD may be a useful TCM for the treatment of POF.	[44]
